# An Integration of a Peristaltic Pump-Based Extruder into a 3D Bioprinter Dedicated to Hydrogels

**DOI:** 10.3390/ma13194237

**Published:** 2020-09-23

**Authors:** Dorota Bociaga, Mateusz Bartniak, Krzysztof Sobczak, Karolina Rosinska

**Affiliations:** 1 Division of Biomedical Engineering and Functional Materials, Institute of Materials Science and Engineering, Lodz University of Technology, 90-924 Lodz, Poland; mateusz.bartniak@poczta.fm (M.B.); rosinska.karolina94@gmail.com (K.R.); 2 Institute of Turbomachinery, Lodz University of Technology, 90-924 Lodz, Poland; krzysztof.sobczak@p.lodz.pl

**Keywords:** 3D bioprinting, extrusion, sodium alginate/gelatin, bioink modeling and testing, tissue engineering, bioprinter design

## Abstract

The 3D printing technologies used for medical applications are mostly based on paste extruders. These are designed for high capacity, and thus often feature large material reservoirs and large diameter nozzles. A major challenge for most 3D printing platforms is a compromise between speed, accuracy, and/or volume/mass of moving elements. To address these issues, we integrated a peristaltic pump into a bioprinter. That allowed for combining the most important requirements: high precision, a large material reservoir, and safety of biological material. The system of a fully heated nozzle and a cooled print bed were developed to maintain the optimal hydrogel temperature and crosslinking speed. Our modifications of the bioprinter design improved the mechanical properties of the printouts and their accuracy while maintaining the maximal survival rate of cells and increasing the capacity of the bioink reservoir.

## 1. Introduction

Nowadays, many people are waiting for a transplant and in many cases it is the only method to save their lives, especially when it comes to the most important organs, such as the heart, kidney, or liver. Unfortunately, not enough donor organs are available to fill the need. Simple tube-like tissue structures are already printable, but fully functional organs not yet. However the development of the bioprinting year by year gives more and more hope that organ production for individual patients will be possible. Bioprinting is a biological branch of 3D printing. In this technique the layers of a material are deposited one on the top of the other in order to construct three-dimensional objects one at a time. Machines for direct bioprinting use bioink-printable biomaterials mixed with living cells or biologically active compounds. The most commonly used biomaterials are hydrogels, e.g., sodium alginate and/or gelatin. Such mixtures are meant to provide high rate of cell reproduction and good printability. Apart from biocompatibility the hydrogel’s viscosity is an important aspect also, as it influences not only the printing process (printability) but also cell proliferation [1,2].

There are several printing techniques. The most popular is the extrusion-based bioprinting (EBB), where bioink is pushed through a nozzle attached to a print head. The nozzles diameters are rarely wider than 400 µm. This is to provide material strain thin enough to build complex geometries and thick enough not to damage cells suspended in the bioink by extrusion pressure being too high and/or too small capillary diameter [3,4,5]. Furthermore, this way of material feed is subjected to a range of performance restrictions, often printing at slow speeds and producing only simple geometries. Most fluid-based extruders do not have the retract function which is the reverse movement of the material (reversing extrusion). Non-extruding movements might lead to unwanted leaking or oozing which can be prevented by retracting of the material, thus preventing bad printout results such as low quality, bulging corners, over-extrusion, and defects such as thin, whisker-type strands of material. There are some ideas regarding the possible ways to deal with this problem. For example, the Envision TEC Bioplotter system [6] has the automatic nozzle cleaning depot utilized between subsequent layers during printing. Thanks to this solution, the print quality increases, however, the print time of even small printouts increases significantly. In the case of printing with bioink (direct bioprinting), such an effect is undesirable. Bearing in mind this type of inconvenience, we implemented retraction to our 3D printing system, and therefore it is able to print complex objects without significant oozing of the printing material. As the peristaltic pump delivers material from the reservoir that can be refilled during printing, the system has the potential to print large-volume objects.

Syringe pump extruders that are currently in use are mostly of high resolution or low volume, which is due to a compromise between moving mass and high positioning precision. Thus, commercial bioprinting systems commonly implement 10 mL material reservoirs to reduce carriage mass; this solution limits the printout size, however.

The extruder introduced by Hinton et al., incorporated with the FRESH 3D printing system called the Replistruder, precisely represents the compromise between precision and moving mass of bioprinters [7]. The Replistruder was designed to provide the capability to retract biomaterials, but its ability to print large objects is limited due to the use of maximum 10 mL of printing material reservoir. Replistuder modifications aimed to increase the volume of the syringe, however, additional extra weight put to the carriage may affect negatively printing performance. Compton and Lewis developed a high-accuracy syringe extruder that is pneumatic-driven [8]. It allowed for printing with use of polymer or high-quality epoxy using low-volume material reservoir, but due to the materials used, it cannot be called a bioprinter. Whereas commercial paste extruders, like Fab@Home Scientist Printer [9], PrintrBot Paste and Extruder [10], Discovery Paste Extruder [11] or ZMorph Thick Paste Extruder [12], have 30 to 100 mL syringes as their material reservoirs, but what they gain in volume, they lose in efficiency and precision. Those systems normally operate with nozzles of large diameter. It reduces resolution and makes the printing process less demanding, but at the same time these machines print at low speeds to compensate for their more massive payloads [13,14,15,16,17]. The Bowden extruder approach could be used to reduce excessive mass on moving elements. This type of system connects the material reservoirs to the nozzle via a tube, avoiding a high reduction in speed. Such systems are usually pneumatically driven, but they have some weaknesses, including extrusion pressure, which varies depending on the amount of the material remaining in the reservoir and the need for precise vacuum while printing to allow retraction. Pneumatically driven systems feature also force response delays that depend on both the rheological properties (e.g., Newtonian, shear thinning, thixotropy) of the material being extruded and the volume of the material reservoir. Pneumatically driven systems are also expensive and bulky, as they require a separate air pressure supply, which increases the overall cost, reduces compactness of the system and complicates the entire structure without increasing its usability. In our system that uses a Bowden-style extruder, the piston is moved by a stepper motor, which provides consistent extrusion pressure and retraction.

The scientists all over the world are improving and developing their bioprinters. The rapid development in the bioprinting field and the obvious need in this area is indicated by the number of books and papers on the subject that have been published recently, e.g., “organ printing” being one of the newest [18], as well as scientific reviews such as the one by Murphy and Atala [19]. Similarly to other 3D printing techniques and branches, bioprinting has its adaptations and evolutions as well. One of the examples is the hybrid method of bioprinting, which is a combination of the fused deposition modelling to produce solid biodegradable scaffold with simultaneous bioink bioprinting. This approach was developed by Malda’s group at the Utrecht University (The Netherlands) [20] and later by Atala’s group at Wake Forest University in North Carolina (USA) [21]. Mironov et al. in 2003 introduced a concept of three-dimensional additive manufacturing of organ printing based on tissue spheres as building blocks to overcome the low cell density in bioink [22]. Organovo Holdings Inc. (USA), which formulated the original method of bioprinting tissue with the use of spheroids, has failed to make the technology reproducible as the tissue spheroids usually fused before they were dispensed. The patented know-how was developed by Forgacs’s group at the University of Missouri (Columbia, USA) and Mironov’s group at the Medical University of South Carolina (Charleston, USA). Teams worked on printing tube-like structure adopting various 3D printing techniques. Forgacs’s group and Organovo developed rod-like continuous dispensing, allowing for the vertical tube preparation [23]. Guerra et al. are working on 3D printed biocompatible scaffolds of 8 mm in diameter or less [24]. The bioprinter of their design was based and built on Fused Deposition Modeling (FDM) technique and 3-axis 3D printing technologies. The idea is, that the material passes through extruder nozzle, which deposits the material onto a computer-controlled horizontal rotary shaft forming a print bed. Gao’s group from Zhejiang University (China) uses a similar approach [25]. In their solution a coaxial needle system is applied, where the outer needle provides hydrogel, and the inner needle is used for delivering a crosslinking agent. This process generates the tube-like structures such as printouts with fluidic channels inside the structures. Other teams developed a non-scaffolding approach to 3D printing such as novel scaffold-free “Kenzan” platform technology based on an array of surgical needles for robotic assembly of tissue spheroids. They use a robotic “pick and place” device according to predesigned digital model, which has been introduced and successfully commercialized by Cyfuse Biomedical K.K. (Japan) [26,27]. Another approach to non-scaffolding bioprinting has been represented by three groups including Woodfield’s group at the University of Otago (New Zealand) [28], Zimmermann’s and Koltay’s group from the University of Freiburg (Germany) [29], and Mironov’s group. Another approach is represented by 3D bioprinting Solutions (Russia) [30]. The company has tried to develop extrusion type 3D bioprinters being able to dispense single spheroid at a time using built-in microfluidic device. Groups of Woodfield and Zimmermann and Koltay plan to commercialize this advanced type of extrusion 3D bioprinters, which in combination with previously achieved functionalities, could become a new standard with two main functionalities: 3D bioprinting of biomaterials mixed with living cells and 3D bioprinting of tissue spheroids.

There are several companies providing complex services for 3D bioprinting, including printers, printing materials and technical support [31,32]. Commercial 3D bioprinters cost varies from $10,000 to over $200,000. This type of machines usually does not offer the possibility to make modifications to both hardware and software. All these issues hinder the innovations. That is why this field of research remains mainly at the academic level and used in laboratories to investigate new techniques without application of commercial machines. A lot of input is still needed into the development of bioprinting technology, so that bioprinters can become market products that enable the production of functional tissue structures.

In our work we take into consideration the devices which are already implemented and commercially available or at least being under development. The main aim of our efforts was to design a system, in which we could: (1) minimize the cost and difficulty of its creation by the application of 3D printing techniques into the peristaltic pump manufacturing process; (2) increase the volume of the bioprinter’s ink reservoir; (3) provide a platform for reliable, rapid and easy alteration of printer specifications using basic knowledge of bioink properties. As a result, we developed an inexpensive (about $500 in total for the entire system) 3D bioprinting system with integrated open source peristaltic pump extruder that can retract, operate at moderate speeds, and fabricate complex objects by additive manufacturing method. The bioprinter has a stirred and heated bioink reservoir that can be refilled during printing process. As a result, the system can potentially produce large-volume objects. Furthermore, the system is capable of providing high printing precision and can be used with most of open source desktop 3D printers.

## 2. Materials and Methods

3D printer construction: The mass of moving elements is maximally reduced. The device is capable of retraction, it is inexpensive, and based on an open source firmware. It is designed for easiness of fabrication, installation and operation by the average user. Essential elements of the system were 3D printed. Thanks to this, the construction costs are low and its modification is simple. Non-printed parts include standard hardware such as bearings, M3 and M5 nuts and bolts, and polyurethane tubing with connectors. Printed parts were manufactured with use of ABS 3D printing filament (Wolfix Company, Warsaw, Poland).

For bioprinting purposes the following components of the system were designed and optimized: a peristaltic pump (Section 2.1), a nozzle with simulated flow and its heater (Section 2.2 and Section 2.3), a bioink reservoir (Section 2.4), and a cooled print bed (Section 2.5). Using hydrogels (Section 2.6 biomaterial preparations) and printouts made of them, proposed solutions were tested for printing accuracy (Section 2.7), layer adhesion (Section 2.8) and influence of printing parameters on the viability of cells placed in a hydrogel (Section 2.9).

Extruded material-hydrogel: All tests aimed at calibrating and verifying the correctness and effectiveness of the 3D printer construction were conducted using hydrogels with two different compositions: 5% w/v alginate and 3% w/v gelatin (marked as 5A3G), and 6% w/v alginate and 4% w/v gelatin (marked as 6A4G). Both were dissolved in deionized water (conductivity of 0.07 μS). Sodium alginate (Sigma-Aldrich, Saint Louis, MO, USA), gelatin (Sigma-Aldrich, Saint Louis, MO, USA) and calcium chloride were used as a cross linker (Sigma-Aldrich, Saint Louis, MO, USA). For testing the viability of endothelial cells, line EA.hy926-ATCC^®^ CRL-2922™ (LGC Standards, London, UK) was applied. These materials were used in a second step to verify the introduced modifications of the developed design, which consisted of: peristaltic pump (Section 3.1), flow analysis in the nozzle (Section 3.2), nozzle carrier and heating block (Section 3.3), cooled print bed (Section 3.4), printout accuracy (Section 3.5), layer adhesion (Section 3.6) and cell viability (Section 3.7).

### 2.1. Peristaltic Pump

**Figure 1:**
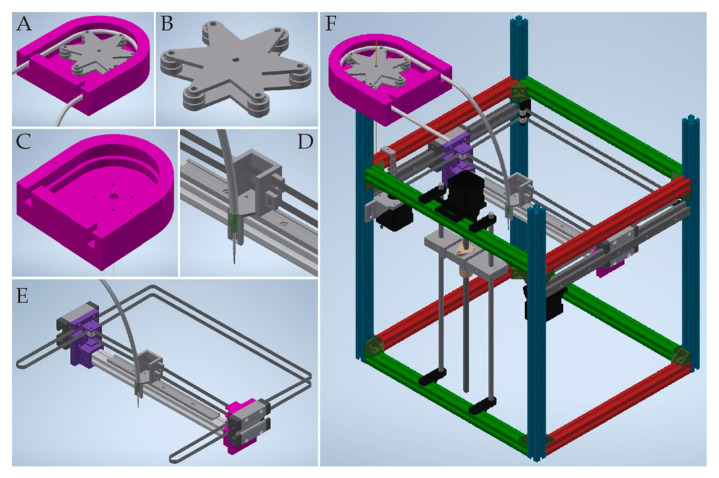
3D printed pump components and the X-axis, where the nozzle is fixed, modeled in CAD (Computer-Aided Design) software. (**A**) assembled peristaltic pump used in the system to extrude hydrogel, (**B**) rotor equipped with rollers, (**C**) core of the pump, (**D**) nozzle holder with heated nozzle and timing belts attachment, (**E**) assembled the X-axis of the printer, (**F**) general view of the 3D printer design.

**Figure 2:**
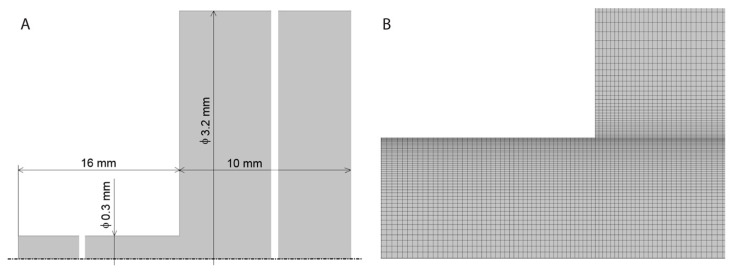
(**A**) A scheme of the flow domain geometry; (**B**) The computational mesh at the nozzle inlet.

**Figure 3:**
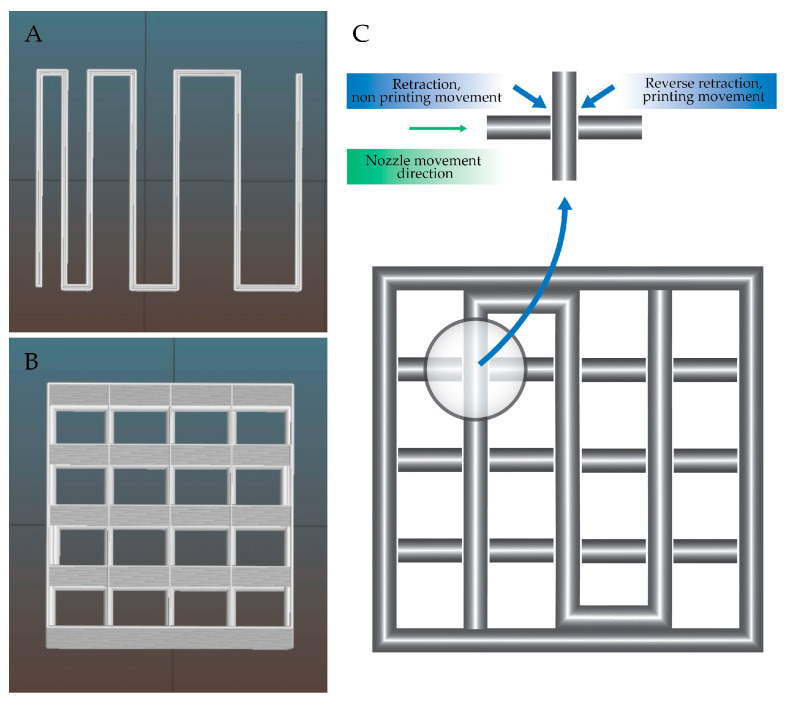
Models for testing a bioink distribution and the accuracy of spatial printouts: (**A**) single line, (**B**) multi-layered grid and (**C**) visualization of how the retraction function was applied to the printed model.

The system consisted of five main parts: (1) the casing of the pump, which supported the hose (Figure 1C), (2) a rotor equipped with rollers moved by the motor (Figure 1B), (3) a hose for hydrogel transportation, (4) a nozzle to extrude material and (5) a nozzle holder (Figure 1D). The designed pump was compatible with an outer diameter of hose equal to 6.4 mm. This size of tubing allowed for proper uniform micro extrusion through needles of various diameters. The constructed system was compatible with needles of many sizes, unlike past extruders that usually use nozzles with a diameter up to 4 mm. To produce printouts, we used a stainless steel blunt tip needle ranging from 300 to 620 µm of inner diameter. Therefore, our hybrid system for providing large-volume extrusion was not subjected to compromise between printout size and its detail that frequently limits commercial syringe extruders.

### 2.2. Nozzle Holder

The system was mostly designed to work with 3D printers with core-XY kinematics possessing linear technology, as it provided the smoothest and the most reliable movement for all sorts of systems. Moreover, such 3D printers had one of the highest structural rigidities. As the moving parts of the system had highly reduced mass, the printer could work with higher speed and acceleration. Accordingly, the carrier could stop fast and with high precision, which led to the best possible printing moves and model mapping. The motion of the motors was transferred to the carrier via a GT2 toothed belt system.

### 2.3. Fully Heated Nozzle with Controllable Flow

The hose ended with a Luer lock to allow for the connection of a nozzle of a specific inner diameter. The nozzle had temperature regulation, so that the viscosity of the hydrogel could be controlled to ensure the optimal extrusion of the bioink. Moreover, the maintained conditions provided a better environment for the cells. Additionally, the heated nozzle allowed for a higher concentration of gelatin which positively stimulated cell proliferation [33]. The nozzle was installed in the heating block. This controlled the temperature of the passing material, and thus continuous, uniform extrusion of bioink with wide range of viscosities and different cells concentrations was possible.

Material extrusion was forced by peristaltic pump operation to provide the pressure rise, which allowed for supplying the bioink at a specific rate to the printing zone. Despite the fact that the flow rate was low, the pressure necessary to overcome flow resistance could be substantial, due to the high viscosity of hydrogels and small diameter pipe use. The key element was the nozzle in the form of a capillary of a very small internal diameter and a significant length. Taking into account that the hose and other elements connecting the pump and the nozzle were of a much higher diameter, their resistance could be neglected.

In order to determine the pressure drop in the nozzle, the exact viscosity of the hydrogel had to be identified. RheolabQC rheometer (Anton Paar GmbH, Graz, Austria) was used to determine the curves of viscosity versus shear rate of the fluids.

In this case, simple relations of the Hagen–Poiseuille flow for laminar flow of Newtonian fluids cannot be applied. Therefore, the simulations of the flow of the hydrogels in the nozzle were performed with ANSYS Workbench 19.2 software (19.2 Release, Ansys Inc., Canonsburg, PA, USA). In order to determine the entrance length of the capillary, the transition between the hose and the nozzle was taken into consideration. The geometry of the numerical model and the computational mesh in the region of the nozzle inlet are shown in Figure 2A,B. The flow was considered two-dimensional, neglecting the component flow in the circumferential direction. The tests of consecutive refinement of the computational mesh allowed for reaching the mesh independent solution.

The steady state flow simulations of 5A3G and 6A4G hydrogels were performed with ANSYS CFX 19.2 solver (19.2 Release, Ansys Inc., Canonsburg, PA, USA). In general the 3D printing processes are transient, but nozzle flows do not vary in time over relatively long periods, so they can be approximated as steady state ones [34,35]. The hydrogels were considered incompressible and isothermal and of constant density (ϱ = 1600 kg/m^3^). Taking into account small nozzle diameters (D = 300 µm), low flow velocity (v_ave_ = 2 mm/s) and high viscosity, the flows were laminar with Reynolds numbers Re= (vaveDϱ)/μ ≤ 2×10−3. In the simulations, the fluid viscosities were defined on the basis of the measurement data shown on the chart presenting flow curves (effective viscosity versus shear rate) for both hydrogels in Section 3.2.

The boundary condition was imposed on the inlet for the mass flow rate which corresponded to the average velocity in the nozzle of v_ave_ = 2 mm/s. Absolute pressure (100 kPa) was applied to the outlet. The no-slip condition was set at the walls.

### 2.4. Bioink Reservoir

A limited bioink reservoir is the one of the biggest problems of the systems currently being used. Our system was specifically designed to solve this issue. The main part of the system was a peristaltic pump, which allowed for extrusion, as well as retraction. The peristaltic pump and the bioink reservoir were placed in a thermal isolated container. Its inside temperature was regulated by the heating plate with thermistor. The reservoir was constantly and gently stirred in controlled environment, kept the specific temperature and could be easily refilled during printing process. The above protected the material against delamination and prevented the cells from falling to the bottom of the reservoir and creating pellets (which would lead to a non-uniform distribution of the cells in a printout). Our system ensured that the biological material was evenly distributed throughout the entire volume of the bioink during the entire printing process. The mixture was collected, transported by pump operation and deposited onto the print bed via the tubing and the nozzle. The whole system provided stable temperature conditions for the tank, hose and the tip of the nozzle to ensure adequate parameters for the cell survival and the hydrogel properties.

### 2.5. Cooled Print Bed

The 3D bioprinter was equipped with a cooled print bed based on a 60 W Peltier cell. The cell size was 40 × 40 mm with a 2 mm copper plate on its top. It had a path for the thermistor drilled in the middle and 2 mm glass on the top of the print bed for improved adhesion between the hydrogel and the print bed surface. All layers were joined with heat-conducting paste with a thermal conductivity of 4.2 W/mK. The print bed size was 35 × 40 mm in dimensions due to the layer change. The system allowed for the instant solidification of gelatin in the hydrogel right after printing.

### 2.6. Bioink Preparation

Bioink was used to check the efficiency of the 3D printing construction in terms of safe extruding possibility of hydrogel material containing living cells. It was prepared according to the method described in our previous paper, where the viability test was conducted [33]. To summarize: sodium alginate, gelatin and calcium chloride were sterilized with UV C light for 60 min. Then 5% weight/volume sodium alginate and 4% w/v gelatin were stirred in Dulbecco’s altered Eagle’s medium (DMEM, Corning Incorporated, Corning, NY, USA) supplemented with 10% fetal bovine serum (FBS) (Corning Incorporated, Corning, NY, USA) and 1% antibiotic penicillin/streptomycin (P/S) (Corning Incorporated, Corning, NY, USA), heating whole system to 37 °C. Endothelial cells, line EA.hy926-ATCC^®^ CRL-2922™ (LGC Standards, London, UK) confluent on level of 80% underwent tripsinization and then were suspended in full culture medium. Prepared cells were centrifuged for 5 min at 180 g. The supernatant was collected and the hydrogel solution was introduced into the cell pellet remaining in the falcon tube. The mixture was manually pipetted until mixed. The bioink was degassed through centrifuging to provide uniform extrusion without any material flow interruptions.

### 2.7. Printout Accuracy Verification

The effects of a cooled print bed and nozzle temperature on 5A3G and 6A4G hydrogels extrudability and printouts accuracy were checked. Different geometries were printed out-single line printout and multilayer grid. The height of the grid ranged up to 11 mm, corresponding with up to 57 layers, and it was dependent on the height of one separate layer and the diameter of the nozzle, which could be altered to the needs of the user. The first layer of the model was printed at a speed of 3.5 mm/s, and subsequent layers were printed at 5 mm/s. The lower printing speed of the initial layer follows good practices in additive manufacturing. It allows to obtain better printout to bed adhesion but it does not affect any of the printout properties. These manufacturing parameters were used for every of our printouts produced.

The retraction function was used per each layer. It was computed automatically in the places of crossing of the lines (Figure 3C) and was performed as follows: when the nozzle makes a printing motion and reaches a perpendicular line, the material is retracted. The nozzle makes a non-printing movement, i.e., it moves up towards the Z axis (it moves above the already printed line). Then it slides down the Z axis and performs reverse retraction and starts a printing movement.

Fabricated objects were measured using calliper with resolution of 0.02 mm. Measurements were conducted in X, Y and Z direction of the sample. A total of five samples were measured three times in each direction. Tests were carried out on a single line print and multilayer grid. Tested model dimensions were: 12.5 × 12.5 × 0.35 mm (XYZ) (Figure 3A) and 13.6 × 13.6 × 11 mm (XYZ) (Figure 3B).

### 2.8. Layer Adhesion Test

**Figure 4:**
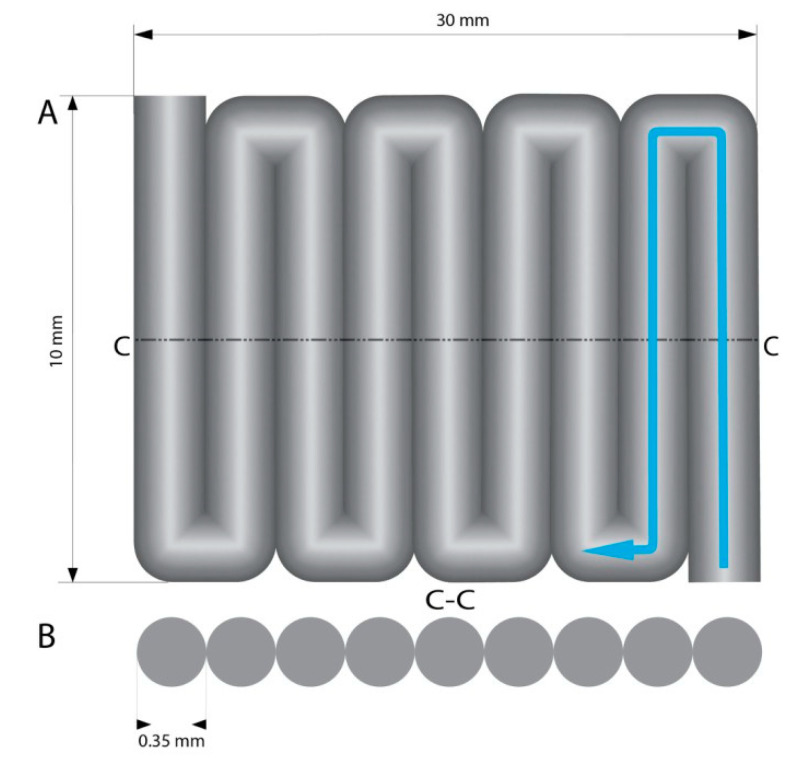
(**A**) Model for layer adhesion testing. Original dimensions: 30 mm long, 10 mm wide, 0.35 mm high. Arrow shows schematic trace of nozzle movement during printing. (**B**) Model cross section view (**C-C**). The diameter of the printed path equals 0.35 mm.

Layer to layer adhesion determines the structural printout strength [36]. 3D printed elements have the lowest durability at the layer connection, so determining the strength of this connection allows us to calculate the strength of the entire model. Printouts for the layer adhesion test were printed as a single layer pattern. The printout lines were printed one next to the other, in such a way that the first line adheres on its whole length to a subsequent line (10 mm single line length) creating the cuboid plate with dimensions 30 × 10 × 0.35 mm with no gaps. The printout geometry was designed in such a way that the printed 10 mm lines were perpendicular to the longer axis of the printout (Figure 4). Printouts were subjected to a tensile test with static velocity of 0.1 mm/s on a tribotester (UMT-2, Bruker, Billerica, MA, USA). At the time of tensile strength test, the model stretched like a harmonica allowing for layer delamination on the layer shift. The tests were conducted five times, and the average value was calculated. The material used for the test was 5A3G.

### 2.9. Viability Test for Cells in Printouts Made of Hydrogels

In order to verify the influence of printing parameters on the viability of cells placed in a hydrogel, the live/dead analysis was performed for cell-laden bioink and for printouts. The entire system (device, wires, print bed and machine frame) was sterilized to ensure antiseptic printing conditions. Prepared printouts (crosslinked with calcium ions) were stained with two dyes diluted in PBS buffer (Corning Incorporated, Corning, NY, USA): calcein and ethidium homodimer (Biotium Inc., Fremont, CA, USA). A staining solution of 4 mM Calcein AM and 2 mM EthD-III was prepared by thoroughly mixing. Samples immersed in the staining solution were incubated in darkness for 30 min. Stained printouts were observed and imaged using a fluorescence microscope (NIKON Eclipse LV100ND, NIKON CORPORATION, Tokyo, Japan).

The quantitative results of the investigated material were statistically analyzed. One-way analysis of variance (ANOVA) was applied. If *p* < 0.05, the results were considered statistically significant.

## 3. Results

**Figure 5:**
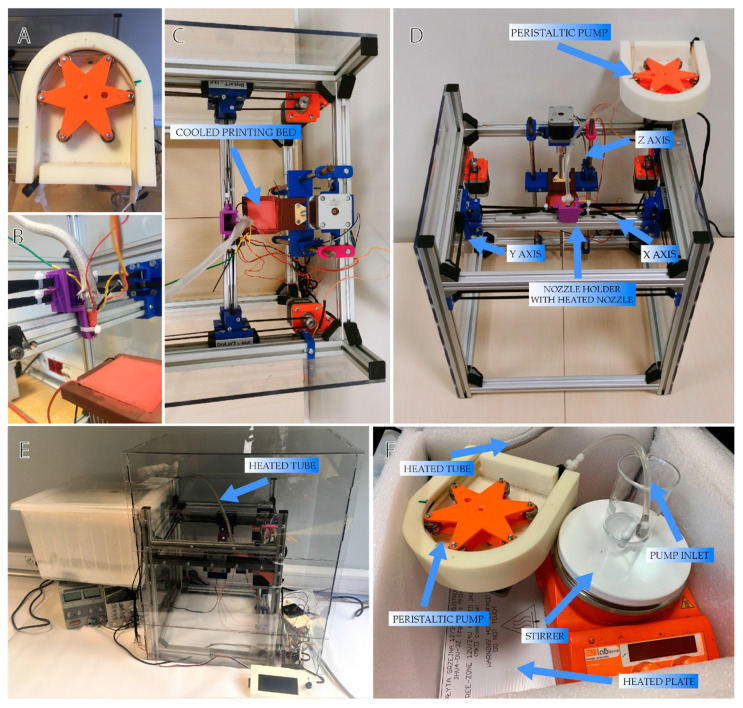
Pictures of the constructed 3D bioprinter with all integrated and tested elements. (**A**) peristaltic pump, (**B**) nozzle holder with heated nozzle and timing belts attachment, (**C**) assembled the X-axis of the printer and cooled printing bed, (**D**) a general view of the 3D bioprinter construction, (**E**) printing system view: pump isolated container and printer “cage”, (**F**) pump isolated container with stirred reservoir and heating system.

In order to check the correct configuration of the constructed system (Figure 5) and adjust the amount of hydrogel administered (with its specific parameters), the tests described below were carried out.

### 3.1. Peristaltic Pump Tests

**Figure 6:**
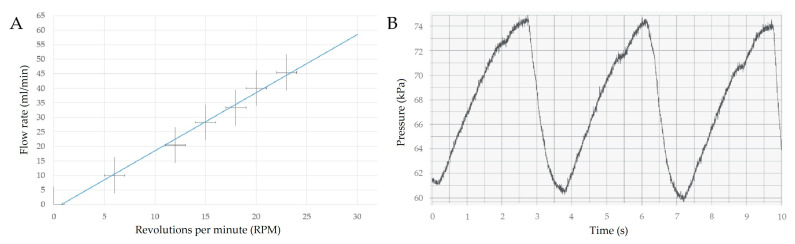
Characteristic of constructed peristaltic pump: (**A**) working speed (error bars are the standard deviation (SD) bars), (**B**) pressure fluctuations during work.

In order to check the performance of manufactured peristaltic pump of our design, the flow rate over time and pressure pulsation during work of the pump were tested. Examinations of the pump behavior allowed for a controlled dosing of the bioink due to proportional increase of the hydrogel being pumped with increase of working speed of the peristaltic pump (Figure 6A). Pressure fluctuation was observed during pump operation. Those alterations were characterized by the repeatability of occurrence in amplitude and intervals (Figure 6B).

### 3.2. Nozzle Flow Analysis

**Figure 7:**
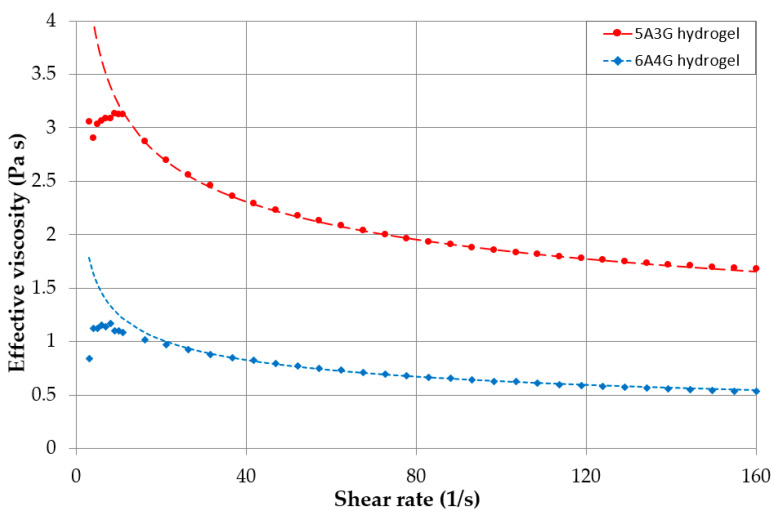
Flow curves (effective viscosity versus shear rate) for 5A3G and 6A4G hydrogels (compositions of alginate and gelatin).

The flow curves for two investigated hydrogels: 5A3G and 6A4G, are presented in Figure 7. Changes of viscosity clearly indicate non-Newtonian fluids. Beside the range of the shear rate lower than 10 s^−1^, the fluids show continuous viscosity drop and can be classified as pseudoplastic (shear-thinning) fluids. Ostwald de Waele model $\left(\mu_{\mathrm{eff}}=k\cdot \gamma^{n-1}\right)$ can be used to approximate the relationship between the effective viscosity $\mu_{\mathrm{eff}}$ and the shear rate γ, where k is the viscosity consistency parameter and n is the power law index. The coefficient values k = 5.6 Pa·s, n = 0.76 and k = 2.5 Pa·s, n = 0.70 for 5A3G and 6A4G were used, respectively to approximate data as shown in Figure 7.

A simple relation of Hagen–Poiseuille for laminar flows of Newtonian fluids could not be applied in this case. Therefore, the numerical simulations of the non-Newtonian fluid flow were carried out with the ANSYS CFX solver according to definition presented in Section 2.3.

**Figure 8:**
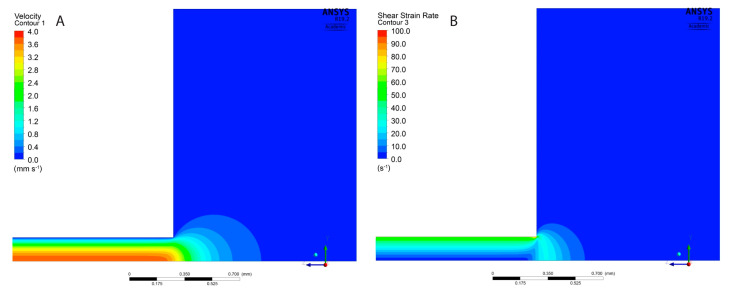
Velocity (**A**) and shear strain rate (**B**) contours at the inlet of the nozzle for 5A3G hydrogel.

**Figure 9:**
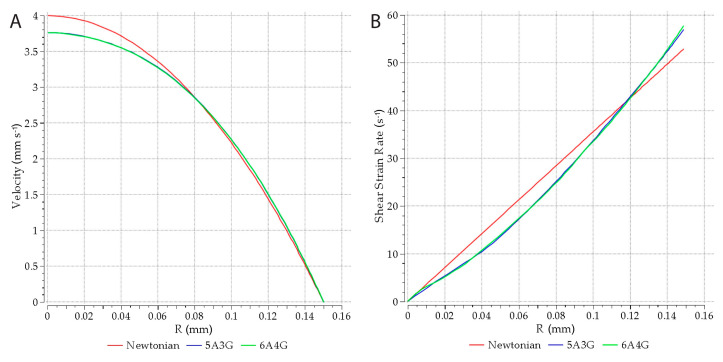
A diagram of fully developed velocity (**A**) and shear strain rate distributions along the nozzle radius (**B**).

The velocity and shear strain rate distributions obtained from the simulations are presented at the nozzle inlet in Figure 8A,B for 5A3G hydrogel. Results for 6A4G hydrogel differed only slightly. They clearly showed that the entrance length in the nozzle was very short. The flow was fully developed (velocity profile shape became constant) at the distance of a half of the nozzle diameter from its inlet. This was in agreement with the entrance length according to relation (*Le* = 0.05 · *D* · *Re*) [37] which, for such low Reynolds numbers as in the cases under analysis, are negligibly short. The fully developed velocity and shear strain rate distributions along the nozzle radius are shown in Figure 9A,B for 5A3G and 6A4G hydrogels as well as for a Newtonian fluid (with the same density, but constant viscosity equal to 0.8 Pa·s). In general, no differences in the distributions for both hydrogels were observed. Additionally, the distributions of velocity for hydrogels did not differ significantly from the parabolic function for Newtonian fluids. However, it was clear that the higher effective viscosity for low shear rates caused a decrease of the maximal velocity of hydrogels at the pipe axis. Similarly, the shear strain rate distribution along the radius differed from the linear function for the Newtonian fluid.

Due to the fact that the entrance length is negligibly short, the pressure of hydrogels decreased linearly along the length of the nozzle. The gauge pressure values at the nozzle inlet (i.e., pressure generated by the pump) obtained from the simulations were equal to 26.3 kPa and 9.4 kPa for 5A3G and 6A4G hydrogels, respectively.

### 3.3. Nozzle Carrier and Heating Block Evaluation

The newly designed and manufactured nozzle carrier and the heating block were tested in order to verify the proper distribution of a bioink and the accuracy of the printouts. The highest level of accuracy was obtained for the printouts conducted in mid-tested temperature of 37 °C. In 34 °C tested 5A3G and 6A4G hydrogels were too dense, which hindered its extrusion causing deterioration in print quality. In 40 °C these hydrogels exhibited too high flowability due to their reduced viscosity caused by the higher extrusion temperature. The measured path dimension of the cross section was enlarged in relation to the intended thickness of the path. This resulted in deterioration in printout quality, as a thick single path extruded on the cooled print bed could not keep its shape. This was due to random leakage of the material from the nozzle.

### 3.4. Cooled Print Bed Evaluation

**Figure 10:**
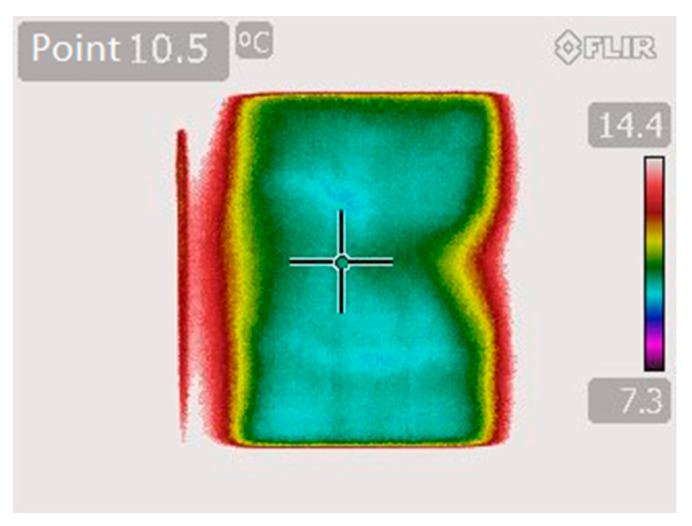
The temperature distribution on the print bed of the 3D bioprinter equipped with Peltier cell.

Thanks to the cooled print bed the printouts did not “spill” outwards and kept their shape with high dimensional accuracy. The temperature ideal for instant solidification of the hydrogel was 10 °C. This allowed for rapid crosslinking of gelatin even up to 14 layers. The system was checked for temperature uniformity on the surface of the print bed (Figure 10). A thermal image was captured with the FLIR T420 Infrared Thermal Imaging Camera.

The temperature distribution is visible as the color map. In the right midpoint of the print bed a higher temperature was observed, which was caused by thermistor path passing between the glass top of the bed and the Peltier cell.

### 3.5. Printout Accuracy Results

**Figure 11:**
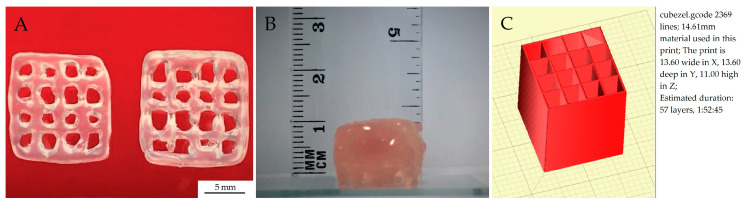
(**A**) Top view of grids printed on peristaltic based extruder machine, used for printout accuracy tests, (**B**) height of the printed model obtained from 57 layers, (**C**) model of the printout with the indicated number of layers.

One of the most important parameters of every 3D bioprinter is printout accuracy. It means that the printed element dimensions are exactly as the predefined CAD geometry. The dimensions of the model were 13.6 × 13.6 × 11.0 mm (X,Y,Z) with 0.28 mm line width (Figure 3B). Results obtained from the printout accuracy test were 13.62 ± 0.11 (mm) for the X axis, 13.63 ± 0.1 (mm) for the Y axis and 10.0 ± 0.5 (mm) for the Z axis (Figure 11). The results obtained depict the repeatability of the printing process, which was influenced by the proper calibration of the printer and printing parameters.

### 3.6. Layer Adhesion Results

**Figure 12:**
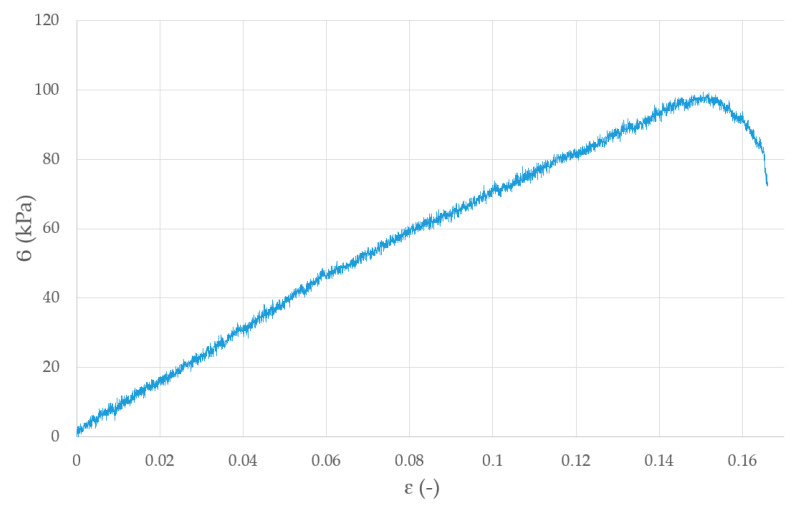
Graph of static adhesion stress for sample 30 × 10 × 0.35 mm for 5A3G hydrogel.

The values obtained from adhesion test were the values of adhesion strength for the single layer-to-layer connection, which is the most important strength parameter of 3D printed elements. Samples printed using our system could withstand a pressure of 98.1 ± 9.06 (kPa) (Figure 12).

The model delaminated on the layer shift under a force of 100 kPa. The layer bonding strength is high enough to withstand forces within the human body, e.g., the stresses exerted on the ureters during urine flow of 20–40 mL/s, are up to 100 kPa [38].

### 3.7. Results for Evaluation of Viability of the Cells after Direct Bioprinting

**Figure 13:**
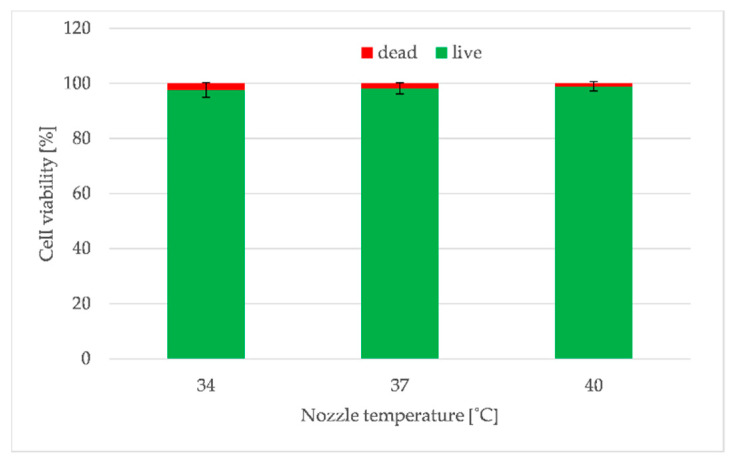
The influence of different temperature of extrusion (34, 37 and 40 °C) on the endothelial cells (EA.hy 926 line) viability of 5A4GM hydrogel. Reproduced with permission from reference [33]. Copyright 2019 MDPI.

The biological study assessed the influence of 3D printer construction (peristaltic pump, temperature stabilized bioink reservoir and tubes, a heated nozzle) on cell viability. The results showed that heating the nozzle above the 37 °C (which is considered the optimal for eukaryotic cells) did not affect cell viability, but could even increase their survival if they were immersed in the bioink. Figure 13 shows the result of cell viability evaluation for printouts produced with nozzle heated to 34, 37 and 40 °C. Although these were not significant differences, one can notice that cell survival was the highest for the nozzle with temperature equal to 40 °C (Figure 13). This was due to the low viscosity of the hydrogels at the temperature of 40 °C, which generated the lowest shear stress as was shown before [33].

The results of cells viability also showed that neither the bioink preparation process nor the different pressure levels (required to extrude the hydrogel at variable temperature) did not adversely affect the cells. Furthermore, the designed solutions of a developed bioprinter, both in the scope of heated tank with mixing function, as well as the entire supply and extruded system of the bioink, provide conditions meeting the requirements for additive techniques for direct printing with the use of hydrogel containing biological material in the form of living cells.

## 4. Discussion

3D printing provides the opportunity to create individual, personalized elements, including those used for medical applications. Additionally, it allows one to make elements from various materials, including thermoplastics, hydrogels and metals.

In this work, both the design of the liquid phase extruder and the printing parameters were oriented towards the bioprinting. In this case, the key issue in the selection of the optimal printing parameters is to determine the main characteristics of the material used for printing, such as its viscosity, density and extrudability. These parameters are in turn directly dependent on the concentrations of components and their temperature. In our case, gelatin and sodium alginate were used in different concentrations to prepare the hydrogel. As we determined the material properties, we were able to select the optimal ranges of the best working parameters of the manufactured liquid phase extruder and the nozzle diameter for deposition of the objects with required accuracy.

The designed system does not add an excessive load to any of the moving elements of the printer. The elements extra weight is put on the printer frame, which does not influence the extruder carriage. This system maximizes printing speed and acceleration because most of the extruder mass is removed from the moving frame. The vibrations of the machine during printing are minimized thanks to this solution. Removing excess mass from the extruder carriage let us also avoid vibration of the lightweight 3D printer, especially when accelerating and decelerating at high speed. Due to this improvement we do not observe printing defects in our printouts such as a “layer shift”—an artifact that influences all subsequent layers and is initiated by stepper motor due to its excessive weight. Additionally, our system may be used with all kinds of open source 3D printers. It was designed for use with the NEMA 17 stepper motors, which can be found on most open source systems. It allows for installation with minimal rework, coding or removal of the parts. A default thermoplastic extruder can be replaced with a nozzle connected to the system via a tube. Furthermore, the system is compatible with a needle size 150 to 800 µm, while available paste extruders mostly use nozzles of a diameter up to 4 mm [9,10,11,12]. In our construction, the needle diameter can be adjusted to the viscosity of the material used and can be easily changed because we use a Luer lock connection applied in a system. Because of performed numerical simulations, the system can calculate proper nozzle diameter and needed pressure adequate to the material viscosity and the temperature of the extrusion. Therefore, in our system we can alter printing parameters even on the level of hydrogel preparation. Additionally, the nozzle flow calculations let us predict if the extrusion pressure required for a specified hydrogel (and expected accuracy of the printouts) is lethal for the cells or not, assuming that fatal pressure for living cells is above 160 kPa [39]. Thanks to all improvements introduced to the system, the parameters can be adjusted before printing to avoid the basic experimental mistakes.

The results of the investigation of the constructed peristaltic pump (Figure 6A) confirmed the proper device operation. Additionally, the measurement of pressure fluctuations showed that these are repeatable (Figure 6B). The tests proved that this device has been designed and manufactured in an appropriate way and meets the requirements for micro-extrusion of high-viscosity materials used as ink in the bioprinting process.

The applied method for cooling of a print bed is similar to the solution in bioprinter BioX6 produced by CELLINK (CELLINK^®^, Gothenburg, Sweden), which is currently one of the world best-selling commercial bioprinters [40]. Introducing a print bed with controllable temperature to our system allowed for the printout quality improvement due to inhibiting the spill effect of the hydrogel without compromising cell survivability. Therefore, it is possible to use not only chemically crosslinked hydrogels but also physically crosslinked ones, which are dependent on the temperature change. The combination of applied designs, together with the ability to adjust the system to the bioink viscosity and the controlled temperature of print bed, creates a system with very high repeatability of printout dimensions. This feature additionally improves machine use in tissue engineering. Moreover, the layer adhesion test showed that bonding strength between each layer of the printouts is at the level of 100 kPa. Because the tensile strength of 3D printed elements is determined by the layer-to-layer connection [41], the improvements of our system to printout durability create elements which can be implemented in a human body.

All commercial bioprinters have very small, volume-limited syringes [31,32,40]. Our 3D bioprinting system has a bioink reservoir that can be refilled during operation and allows stirring and heating of the bioink reservoir, what minimizes cell agglomeration and sedimentation. Thus, the uniform distribution of the cells in bioink is maintained at the same level for the whole printing process. Because the system has a refillable reservoir, it can potentially produce much larger printouts than on other commercial bioprinters while still maintaining a high level of 3D model mapping.

Some bioprinting systems include ultraviolet (UV) lamps for sterilization of the bioprinting chamber [31]. Other systems are based on single use elements [32]. Our system can use both of these methods of sterilization, or can be autoclaved. This is due to the fact that all elements being in contact with the cells are temperature resistant, can be easily dismounted and sterilized. This positively impacts not only the environment, but also the cost of single printout.

An easy, fast and cheap sterilization process of the printing system parts having contact with biological material together with a possibility to apply a refillable bioink reservoir are the main advantages of using peristaltic pump compared to other extrusion based bioprinting techniques.

## 5. Conclusions

Joint efforts at the international level contribute to the rapid development of bioprinting technology, both in the range of materials used in printing and the bioprinters themselves. Direct bioprinting for the use in tissue engineering is subjected to rigorous requirements as the accuracy of the printouts in relation to the CAD model, the survivability of the cells constituting the biological material suspended in the hydrogel and maintaining the sterile conditions during the printer work. In our case, the main emphasis was placed on the device, which allows for efficient and direct bioprinting. We have designed and tested a system that corresponds with important needs.

The controllable temperature of the system elements is one of the most important issues to ensure the safety of the cells and suitable properties of a bioink. Our system was designed to provide an extrusion temperature selection in the range of 30–40 °C. Although 37 °C is considered the optimal temperature for eukaryotic cells, the results showed that heating the nozzle above this limit can be a very valuable feature of the extruder. Bioink warmed up to 40 °C during extrusion does not affect cell viability and can even increase their survival as the elevated temperature decreases hydrogel viscosity and shear stress and thus decreases the pressure needed for extrusion.

In order to achieve an appropriate accuracy of the printouts, the nozzle positioning and extruded bioink volume are crucial. Pump characteristics helps to adjust it to the extrusion conditions. Refining and adjusting the printer parameters such as extrusion speed, viscosity of bioink, nozzle diameter, the temperature of the hydrogel and the temperature of the cooled print bed provides proper printouts by extruding the designed amount of material into defined shape on the print bed. Incorporation of the Peltier cooled print bed extends the range of materials that can be used in the system, as it allows for an instant physical crosslinking of thermocurable hydrogels such as mixes containing gelatin.

Numerical simulations of the hydrogel flow in the extruder allow one to easily test the flow conditions, and adjust nozzle design to the specific material viscosity. As the whole system is modeled in CAD software, the nozzle can be easily modified to ensure that the provided material is properly extruded, and the internal pressure in the hose does not exceed 160 kPa, which is the maximum pressure to maintain the cells proliferation capability.

All elements were designed to create a printer that meets the requirements of 3D printing for tissue engineering. Solutions proposed in our system can contribute to the progress in the clinical use of bioprinting. It can also accelerate the actual introduction of bioprinting process and techniques to human tissues reproduction. All the solutions designed for our bioprinter system can have very valuable features for additional applications besides tissue scaffolds and medical applications. Using them in any other 3D printers can make the process of testing new materials much cheaper and faster.
